# Rewarming: facts and myths from the systemic perspective

**DOI:** 10.1186/cc11283

**Published:** 2012-06-07

**Authors:** V Scaravilli, D Bonacina, G Citerio

**Affiliations:** 1 Department of Experimental Medicine, University of Milano-Bicocca, Ospedale San Gerardo, Monza, Italy; 2 Neuroanesthesia and Neurointensive Care Unit, Department of Perioperative Medicine and Intensive Care, Ospedale San Gerardo, Monza, Italy

## Introduction

Rewarming is a delicate phase of therapeutic hypothermia (TH). Adverse consequences of rewarming on the whole body may seriously limit the protective effects of hypothermia, leading to secondary injury. Thus, understanding, predicting, and managing possible systemic side effects of rewarming is important for guaranteeing TH efficacy. The aim of this brief report is to describe rewarming effects from a systemic perspective.

## Hemodynamics and imbalance in oxygen consumption and delivery

TH linearly decreases the metabolic rate of homeothermic organisms. During the cooling process, tissue oxygen consumption (VO_2_) slows by roughly 6%/°C reduction in body temperature [1,2], obeying the van't Hoff-Arrhenius law, which states that the rate of a biochemical reaction is halved for each 10°C decrease in temperature. The reduction in brain metabolism is similar [3].

In contrast, during rewarming, the possible appearance of a mismatch between total body oxygen demand and oxygen delivery (DO_2_) [4] has been recognized since the pioneer works of Hegnauer and colleagues on dogs [5] and Bigelow on humans [6]. Bigelow has described this side effect of rewarming as *rewarming shock*: 'This syndrome of acute acidosis, or rewarming shock, was characterized by a progressive decline in blood pH [...] associated with respiratory inadequacy [...]. A fall in blood pressure and tachycardia were features in some cases'. In more recent studies, rewarming shock after moderate TH seems to be a more infrequent eventuality, probably because TH management has been completely changed by the advent of ICUs and a far less hypothermic regimen. The mismatch between oxygen supply and consumption during rewarming could depend on numerous factors, including metabolic rate, abnormalities in oxygen extraction, cardiac output (CO), circulating blood volume, regional blood flow, pH, blood viscosity, and a shift in the hemoglobin dissociation curve. The physiopathology of this side effect of rewarming is not known. Rewarming from hypothermia is such a complex and metabolism-pervasive process to alter all of the possible determinants of a VO_2_/DO_2 _mismatch. Of the possible determinants of VO_2_/DO_2_, cardiac dysfunction has been the most investigated. Cooling determines a proportional decrease in cardiac output [1], heart rate, and mean arterial blood pressure, with no change in stroke volume and increased peripheral vascular resistance. During the maintenance stage of TH, the decrease in metabolic rate is equal to or greater than the decrease in cardiac output, and alteration of oxygen delivery is not a matter of concern. Preliminary clinical studies [7] and a recent meta-analysis [8] have shown a decrease in myocardial ischemic injury. Many of the alterations in the cardiovascular system occurring during hypothermia completely reverse during rewarming. Therefore, the rewarming phase could lead to a permanent deterioration of myocardial function and cardiac output. The pathophysiological mechanism underlying cardiac dysfunction induced by hypothermia rewarming has been studied by Tveita's group, first *in vitro *using a rat left ventricular papillary muscle [9] and then *in vivo *[10] in an intact rat model. These studies showed how post-rewarming systolic left ventricular dysfunction can be related to decreased myofibrillar Ca^2+ ^sensitivity due to increased troponin C phosphorylation.

In addition, Blair and colleagues [11] and Morray and Pavlin [12] documented an increase in total oxygen consumption to values above prehypothermic controls in a dog model of rewarming after deep hypothermia. The authors suggested many possible explanations for this event. First, heterogeneous blood flow distribution [13] during hypothermia may determine areas of oxygen debt, with decreased or absent perfusion, that become hypoxic and generate lactate. During rewarming, these areas are reperfused and lactate re-enters normal oxidative pathways, consuming oxygen in the process. Second, with a return to normothermia, free radical oxidation [14,15] and inflammatory response to injury [16,17] could resume, leading to nonrespiratory utilization of oxygen and an increase of VO_2 _over pre-injury control. Third, shivering can occur during rewarming as a response to deviations from the temperature set point. The shivering response to maintain a constant core temperature is a concerted reaction involving skeletal muscle contraction and peripheral vasoconstriction. When shivering occurs during rewarming, it is associated with increased VO_2 _[18,19] and hemodynamic instability [20].

Cain and Bradley 21] and Schumacker and colleagues [22] have described abnormalities of peripheral oxygen extraction in dogs during hypothermia, even with adequate oxygen delivery. An alteration in the temperature transition of oxidative phosphorylation has been documented in an animal model. Leducq and colleagues presented evidence for an abnormal pattern of oxidative phosphorylation control that correlated with a transition in mitochondrial permeability and persisted after rewarming [23]. This phenomenon may cause alterations in oxygen utilization during and after rewarming.

Kondratiev and colleagues addressed the problem of oxygen supply in a rat model of deep hypothermia (15°C) and rewarming [24]. The experiment demonstrated a reduction in cardiac output and oxygen delivery after prolonged deep hypothermia (15°C for 5 hours) compared with less prolonged exposure. The rewarming-related rightward shift of the oxygen hemoglobin saturation curve, which facilitates oxygen dissociation at the tissue level, compensated for compromised peripheral oxygen transport, leading to a stable oxygen supply. Knowing the events causing VO_2_/DO_2 _mismatch during rewarming is important in this phase of TH for monitoring and assuring adequate cerebral and whole body oxygen delivery. Low oxygen delivery accounts for the development of secondary injury, which limits the safety and effectiveness of TH. With this perspective in mind, we can suggest various measures to limit VO_2_/DO_2 _mismatch during rewarming.

First, rewarming after TH should be done slowly and in a controlled manner [25]. Eshel, in a rat model of TH, showed how rapid rewarming from moderate hypothermia is associated with more acute hemodynamic alterations compared with slow rewarming [25]. Similar effects were described in humans [26] and pediatric patients [27] undergoing TH for hypoxic ischemic encephalopathy and deep intraoperative hypothermia (27°C), respectively, as well as in the work of Hanhela and colleagues [28] on adults undergoing cardiopulmonary bypass for cardiac surgery.

Second, controlling pain, sedation, and preventing shivering should limit oxygen consumption. Michenfelder and colleagues [29], Rodriguez and colleagues [30], and Zwischenberger and colleagues [31] demonstrated that the suppression of shivering by neuromuscular blockade is an effective method for diminishing VO_2_. More recently, Badjata and colleagues [32] proposed a simple shivering grading tool, the Bedside Shivering Assessment Scale (BSAS), developed by assessing the correlation of bedside shivering and systemic metabolic stress quantified by indirect calorimetry. Using clinical observation of muscle involvement, the BSAS provides an accurate representation of shivering-related oxygen consumption. Accurately defining shivering intensity assures the possibility of a stepwise treatment for shivering. We recommend initially managing shivering with nonsedating interventions, such as correcting hypomagnesemia, or a serotonin (5-TH) 1A partial agonist like buspirone or meperidine. Meperidine has been demonstrated to effectively reduce VO_2 _augmentation associated with postoperative shivering at a dosage that does not cause respiratory depression [33]. When these first line interventions fail, sedation with short-acting sedative agents and neuromuscular blockade can be used.

Third, oxygen content and transport should be optimized. Anemia and arterial desaturation must be avoided during rewarming. To date, no clinical trials have examined hemodynamic optimization in patients that have undergone TH, least of all during rewarming, and no evidence is currently available to indicate the best strategy for hemodynamic support in such a critical phase. We suggest a strict control of hemodynamics, with the aim of guaranteeing adequate oxygen delivery and avoiding VO_2_/DO_2 _mismatch, using at least continuous arterial pressure monitoring, volume balance and urine output surveillance, and frequent serum lactate measurements. In the case of hemodynamic instability, advanced monitoring capable of finer management could be useful. Thus, in this context, echocardiography and goal-directed hemodynamic optimization [34] may have a place. Treatment of systolic left ventricular impairment presents additional concerns. Pharmacological therapy with catecholamines presents substantial limitations [35,36], as the decreased myofilament Ca^2+ ^sensitivity during rewarming significantly diminishes β-adrenoceptor effects. In addition, catecholamines determine elevated myocardial oxygen consumption and arrhythmogenesis. A recent study by Rungatscher and colleagues [37] tested the efficacy of levosimendan in improving myocardial dysfunction after rewarming from deep hypothermia in a rat model. Levosimendan, as a Ca^2+ ^sensitizer, demonstrated better inotropic and lusitropic effects than epinephrine.

## Glycemic homeostasis

Animal models have shown that hypothermia induces alterations in blood glucose homeostasis via several mechanisms: reduced glucose utilization [38], decreased endogenous insulin secretion [39-41], and increased resistance to exogenous insulin [42,43] . In a recently published prospective observational study dealing with glycemic homeostasis during TH after cardiac arrest (CA), Cueni-Villoz and colleagues found a significantly higher mean blood glucose concentration, blood glucose variability, and insulin dose during TH compared with the normothermia that follows passive rewarming [44]. Because the doses of adrenergic agents did not change significantly between the two steps, the authors advocated lower endogenous insulin levels and the development of insulin resistance as an explanation for the findings. The improvement in glycemic control observed during normothermia, despite lower insulin infusion, suggests that progressive recovery towards normal glycemic homeostasis occurred during rewarming. The rate of hypoglycemic episodes correlated with poor neurological outcome and was similar during TH (8%) and normothermia (7.5%), but more frequently in patients who presented with higher blood glucose variability during TH. These data highlight the importance of progressive tapering of insulin doses to avoid hypoglycemia during passive rewarming from TH after CA, especially for patients who exhibited abrupt glucose shifts during TH.

The importance of glycemic control is further outlined in a recent work by Smith and colleagues. The alteration of blood glucose homeostasis is associated with increased ICU morbidity and poor outcome [45]. Passive rewarming from TH increases insulin sensitivity, but active rewarming from cardiopulmonary bypass decreases it. In both settings, rewarming is characterized by a dynamic insulin/glucose ratio; glucose should be checked frequently and insulin requirements promptly adapted to achieve optimal glycemic control.

## Electrolytes

Mild hypothermia shifts potassium inside the cells and predisposes the patient to hypokalemia, as well as hypocalcemia, hypomagnesemia, and hypophosphatemia. During rewarming, rebound increases in these electrolytes (particularly potassium) may occur, especially if they were replaced excessively during the cooling period [46]. Hyperkalemia can be prevented by slow and controlled rewarming, allowing the kidney to excrete the excess potassium. In patients with severe oliguria or anuria, renal replacement therapy should be started before rewarming to avoid hyperkalemia.

## Systemic inflammation

TH has been shown to suppress ischemia-induced cerebral and systemic inflammation after traumatic brain injury (TBI) in preclinical [47-51] and clinical settings [16]. Following CA and reperfusion, TH is the only effective therapy for increasing survival and decreasing morbidity [52], probably by impairing harmful inflammatory reactions, which characterize systemic ischemia-reperfusion syndrome [53]. In a recent study, Bisschops and colleagues measured the kinetics of inflammatory mediators during TH and rewarming after CA [16]. Proinflammatory IL-6 increased during the TH phase, but values were surprisingly lower after rewarming. Anti-inflammatory IL-10 and IL-1RA did not significantly change over time. Complement and adhesion molecules, an index of endothelial activation, were elevated at admission, fell to low values during TH, and increased again after rewarming, confirming the hypothesis that inflammatory processes reactivate with the increase in temperature [17]. Interestingly, no significant differences were found between artery and jugular samples, confirming that the ischemia-reperfusion phenomenon is not confined to the brain, but affects the whole organism.

Fast rewarming rates have been shown to predict worse outcomes in animal models [49,54,55] due to the rapid reactivation of the inflammatory processes that were set-off by TH. Even if experimental evidence shows the advantages of controlled rewarming, additional clinical studies are needed to determine the optimal rewarming rate and strategy. Several drugs have also been tested recently in cell culture, tissue, and animal models to check their ability to mitigate the detrimental effects of rewarming. Data from Schmitt and colleagues suggest that pretreatment with methylprednisolone increases cerebral cell survival after deep hypothermia [50], but also suppresses important neuroprotective and regenerative processes induced by the proinflammatory cytokine IL-6. Diestel and colleagues focused on endothelial cells [56], which maintain systemic inflammation via cytokine production. Only combined pretreatment with methylprednisolone and tacrolimus inhibited IL-6 secretion. A specific p38 inhibitor was demonstrated to downregulate the unwanted release of IL-6 after cooling and rewarming most effectively. In a rat model of intestinal ischemia, gradual rewarming and administration of dexamethasone improved [48] survival and attenuated ALI after intestinal ischemia/reperfusion injury treated with TH in rats. Alva and colleagues [15] found that metabolic acidosis induced by rewarming was prevented by fructose 1,6-biphosphate (F1,6BP) administration in rats. F1,6BP also protected against oxidative stress induced after rewarming by decreasing lipid peroxidation in the plasma and potentiating antioxidant enzyme activities in erythrocytes. These results may be due to an increase in plasma nitric oxide and leukocytosis after the F1,6BP bolus. Other preclinical studies dealing with the anti-inflammatory safety and efficacy of these drugs are needed before a clinical study can start.

## Infections

The most described infectious complication during TH is pneumonia. As most of the pneumonia diagnoses are made during rewarming or after achieving normothermia [57,58], several authors have claimed that rewarming itself should be considered a high risk for infection. However, as the studies cited above adopted TH for 24 hours and achieved normothermia in the following 12 to 24 hours, the occurrence of pneumonia diagnosis during or after rewarming could also be due to latency from inoculation to the clinical manifestation of infection. Moreover, as hypothermia causes an impaired inflammatory response, clinical signs of infection leading to the diagnosis may be fully detectable only after reactivation of the immune system during or after rewarming. Whether more gradual controlled rewarming can reduce the frequency of pneumonia is unclear. In a small case series [59], even very slow controlled rewarming (0.1°C/hour) was associated with a high frequency of pulmonary infection, perhaps because slow rewarming prolongs the total duration of hypothermia.

## Coagulation

Mild platelet dysfunction occurs at temperatures <35°C, and some inhibition of the coagulation cascade develops at temperatures <33°C. In TH after TBI [58] and stroke [57], the platelet count can also decrease, which persists during and after rewarming. In neonatal cold injury, death occurring during rewarming has been attributed to massive thrombosis from platelet hyperaggregation [60].

All of these data suggest that, in the clinical setting, attention must be paid to rewarming rates and attaining the target temperature to assure the optimal effects of hypothermia. The rewarming rate is an important variable; slower rewarming rates should be routinely employed to avoid systemic side effects.
